# Extracellular Vesicles as “Very Important Particles” (VIPs) in Aging

**DOI:** 10.3390/ijms24044250

**Published:** 2023-02-20

**Authors:** Cristina Mas-Bargues, Matilde Alique

**Affiliations:** 1 Grupo de Investigación Freshage, Departamento de Fisiología, Facultad de Medicina, Universidad de Valencia, Centro de Investigación Biomédica en Red Fragilidad y Envejecimiento Saludable-Instituto de Salud Carlos III (CIBERFES-ISCIII), Instituto Sanitario de Investigación INCLIVA, 46010 Valencia, Spain; 2 Departamento de Biología de Sistemas, Universidad de Alcalá, Alcalá de Henares, 28871 Madrid, Spain; 3 Instituto Ramón y Cajal de Investigación Sanitaria (IRYCIS), 28034 Madrid, Spain

**Keywords:** aging, age-related diseases, biomarkers, extracellular vesicles, inflammaging, senescence, therapeutic agents

## Abstract

In recent decades, extracellular vesicles have been recognized as “very important particles” (VIPs) associated with aging and age-related disease. During the 1980s, researchers discovered that these vesicle particles released by cells were not debris but signaling molecules carrying cargoes that play key roles in physiological processes and physiopathological modulation. Following the International Society for Extracellular Vesicles (ISEV) recommendation, different vesicle particles (e.g., exosomes, microvesicles, oncosomes) have been named globally extracellular vesicles. These vesicles are essential to maintain body homeostasis owing to their essential and evolutionarily conserved role in cellular communication and interaction with different tissues. Furthermore, recent studies have shown the role of extracellular vesicles in aging and age-associated diseases. This review summarizes the advances in the study of extracellular vesicles, mainly focusing on recently refined methods for their isolation and characterization. In addition, the role of extracellular vesicles in cell signaling and maintenance of homeostasis, as well as their usefulness as new biomarkers and therapeutic agents in aging and age-associated diseases, has also been highlighted.

## 1. Extracellular Vesicles: From Cellular Debris to Essential Particles in Cellular Communication

Extracellular vesicles (EVs) are membrane-bound particles (diameter 30 nm to 5 µm) released by almost all cell types [1]. EVs are considered “very important particles” (VIPs) (a new term defined by the authors of this review) in living organisms because of their role in cellular communication, as they carry bioactive proteins, lipids, and nucleic acids as part of their functional cargo [2,3,4]. EVs regulate signaling processes and play key roles in aging and age-associated diseases [3,5,6,7]. EVs are expressed abundantly in body fluids, mainly blood, urine, and saliva [8]. Therefore, EV analysis in body fluids has been proposed as a non-invasive method for the identification of a novel biomarker for many diseases [9,10,11].

The first evidence of the presence of EVs was in the last century. In 1946, Chargaff and West [12] reported some procoagulant particles in plasma derived from platelets, and in 1967, Wolf [13] named them “platelet dust”. In the 1980s, Harding et al. [14] described a new cell-to-cell communication pathway mediated by particles carrying bioactive molecules that could act through autocrine (the cell itself), paracrine (neighboring cells), and endocrine (distant cells) signaling [1,15]. In the late 2000s, EVs were described as vehicles transporting biological molecules between cells, generating significant research interest because of their biological implications [1,16,17]. In the last decade, studies have shown that EVs play a fundamental and evolutionarily conserved role in cellular communication via the trafficking of bioactive molecules from origin cells to target ones. This well-orchestrated communication system is crucial for the organism to respond to external cues in a coordinated manner. Therefore, EVs are intercellular communicators essential for cell homeostasis [17,18,19].

In 2018, the International Society for Extracellular Vesicles (ISEV) updated the guidelines released in 2014 by focusing on the need for appropriate nomenclature in reporting EVs to standardize the protocols and reports in the EV field [20]. The 2018 guidelines for studies on extracellular vesicles (Minimal Information for Studies of Extracellular Vesicles 2018; MISEV2018) recommended the term “EV” as the best generic terminology for the particle constituted by a lipid bilayer that is released by cells [20]. “EVs” is considered a collective term covering various subtypes of cell-released membranous structures including exosomes, microvesicles (previously also known as MPs), ectosomes, oncosomes, apoptotic bodies, and others [21], regardless of their biogenesis mechanism, shedding, molecular markers, size, density, and function [17,21]. The MISEV2018 initiative standardizes the terminology, sample collection and pre-processing, EV separation and concentration, characterization, functional studies, and reporting requirements/exceptions [22].

EV terminology has changed over the years [15,20]. EVs encompass various types of vesicles, including:

(1) Exosome (30–100 nm, the smallest extracellular vesicle) formation and release occur through the endosomal pathway and into the extracellular medium after fusion with the plasma membrane. Its content corresponds to that existing in the endosomal compartment [23].
(2) Ectosomes (100–350 nm) are vesicles found everywhere in organisms and released from the plasma membrane. Their function is analogous to exosomes [24].
(3) Microvesicles (MVs; formerly called microparticles or MPs) have a size from 100 nm–1 µm. They are secreted outside the cell by the process of evagination or sprouting of the plasma membrane, which involves: (a) relocation of phospholipids in the outer membrane so that the phosphatidylserine (PS), generally located on the inner side of the membrane, is exposed on the surface of the vesicle, (b) rearrangement of the cytoskeleton, (c) generation of the curvature of the membrane, and (d) liberation of the vesicle [25,26,27].
(4) Apoptotic bodies (1–5 µm) are released as vesicles after cellular apoptosis, followed by increased membrane permeability, DNA fragmentation, and changes in mitochondrial membrane potential. Apoptotic bodies also expose PS on their surface and contain cellular organelles and genetic material [26,28,29].

Moreover, in the case of large and small EV release by cancer cells, the terminology assigns the term “oncosomes” [30]. Thus, cancer-derived exosomes, ectosomes, and microvesicles are considered “oncosomes” [31].

The EV cargo’s composition reflects the parent cell’s characteristics [1]. For this reason, EVs released by tumor cells can be used as cancer biomarkers. To expand EV applicability, several studies have focused on their potential as biomarkers of aging and age-associated diseases [11,32]. Moreover, EVs’ surface has a specific set of receptors that determine their target cell [33]. Thus, EVs have a specific parent cell and a specific target cell. This feature makes them very helpful for therapeutic purposes and drug-delivery vehicles [11].

Recently, new EVs have been described, “supermeres” and “exomeres.” Zhang et al. [34] identified a new type of bioactive molecule-carrying vesicle called “supermeres,” which can carry cargo (enzymes, proteins, and nucleic acids) associated with cancers, cardiovascular diseases, Alzheimer’s disease, and COVID-19. They are entirely different from EVs; supermeres are considered nanoparticles. These particles are a membranous extracellular nanoparticle type of 15–25 nm and contain RNA and proteins associated with EVs [35]. Supermeres are of great interest because they could be used as therapeutic targets and biomarkers for diseases, thereby improving early diagnosis or targeted treatments. Clancy et al. [36] also highlighted the discovery of supermeres in physiology and disease because of their promising role in clinical translation. Similarly, another new type of non-membranous nanovesicle has recently emerged: the exomeres [37]. These are defined as non-membranous nanovesicles with a size range below 50 nm. 

**Figure 1:**
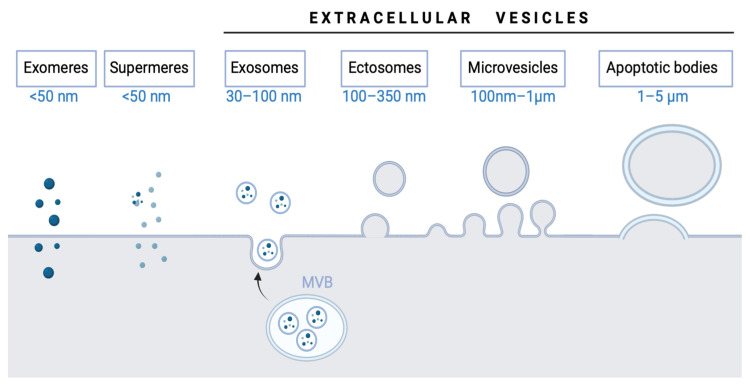
Extracellular vesicle types and subtypes.

Taken together, the extracellular vesicle family is constantly growing and expanding and each of its members is starting to be more accurately defined (Figure 1). Nonetheless, more efforts are still needed before their clinical use.

## 2. Methods of EV Isolation and Characterization

**Figure 2:**
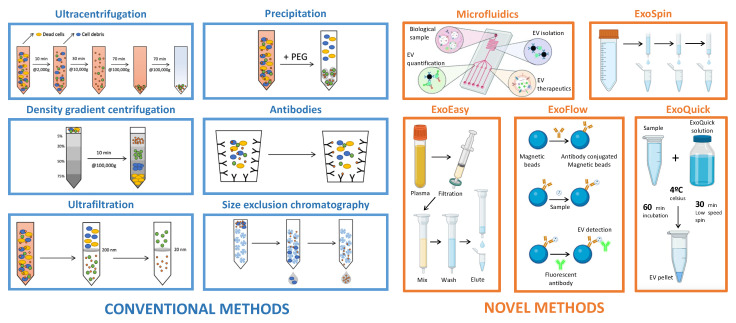
Extracellular vesicle isolation methods.

The scientific community has attempted to develop optimized methods for EV isolation and characterization, increasing the EV nomenclature’s complexity. Konoshenko et al. [38] summarized the classical and recently developed state-of-the-art EV isolation methods, including each technique’s advantages and disadvantages. The main limitation identified in their review was the challenge of developing a universal approach for EV isolation. Commonly used EV isolation methods include ultracentrifugation, density gradient ultracentrifugation, filtration, and techniques based on changes in EV solubility and/or aggregation, size exclusion chromatography, and polymer-based precipitation [38,39]. Novel methods of EV isolation include a two-phase system with polyethylene glycol (PEG) and dextran [40], which utilize affinity-based interactions, commercial kits such as ExoEasy, ExoSpin, ExoFlow, and ExoQuick plus [41], and microfluidic devices (ExoChip) [42]. Both conventional and novel methods for EV isolation are illustrated in Figure 2. Moreover, MISEV2018 describes various separation and concentration methods of EV isolation so that researchers can select the most appropriate methods according to downstream purposes. Thus, to date, MISEV2018 remains the only standardized repository of established methods for EV isolation and characterization [21].

Since 2015, several efforts have been made worldwide to establish and refine EV quantification and characterization methods. In the absence of one optimized and suitable approach, at least two different techniques need to be used to quantify and/or characterize the EV morphology, biocomposition, and receptors [43]. Table 1 summarizes the main EV quantification and characterization methods and their advantages and disadvantages. The selected methods of EV isolation, quantification, and characterization can affect the interpretation of results. Therefore, it is critical to unify them to enable the comparison of studies from different researchers. For instance, researchers must standardize the EV isolation and quantification methods. EVs can be isolated from biological fluids, such as plasma and urine, but they can also be obtained from cells cultured in vitro. In both cases, the many different EV isolation and characterization techniques can lead to differences in EV number, yield, recovery, and function.

## 3. Extracellular Vesicles as Biomarkers in Aging and Age-Associated Diseases

Aging is the main cause of disease and death in developed countries (“Rejuvenome Project,” [52]). Aging is a complex process that progressively compromises most biological functions of living organisms, thereby gradually reducing their quality of life [44]. Most of the compromised functions associated with aging are due to the nine hallmarks of aging, including cellular senescence and altered intercellular communication [45]. EVs regulate both processes. Current knowledge on EVs derived from senescent cells has demonstrated that they are crucial in the aging microenvironment and age-related disease incidence and progression [48]. An increased number of circulating EVs are produced from senescent cells during the progression of aging or age-associated diseases [7,45]. Therefore, EVs can be postulated as an essential contributor with a geronic role in the development of biological and pathological aging. 

### 3.1. Types of Senescence

Some authors considered that biological aging is a disease. Other authors described aging as a natural and universal process, not a disease (defined as a deviation from the normal state) [53]. To date, two types of senescence have been described: senescence-associated secretory phenotype (SASP) and stress-induced premature senescence (SIPS) [3]. While the elderly are mainly associated with SASP, pathologies due to aging appeared in SIPS [54]. Some therapeutic (also known as senolytic and senomorphic) drugs have been developed to avoid or delay the regular/normal cell phenotype switch to senescent cells [55].

Telomerase activity is the main difference between both types of senescence, SASP and SIPS. On the one hand, SASP is characterized by decreased telomerase activity. Therefore, telomere length shortens [56,57,58]; however, SIPS is independent of telomerase and is considered a reversible senescence [59]. Studies in recent years have highlighted that once cells change their phenotype and acquire senescent characteristics, regardless of inducer or stressors, SIPS cells are irreversibly growth-arrested [60]. Despite that, notably, other studies described that during the development of cellular senescence, the activity of the p53 oncogene decreases over time. Therefore, p53 expression and activity play a crucial role in the induction of the senescent phenotype and the constitutive dynamic development of cellular senescence in which p53 activity is induced in the first steps of senescence (primary senescence), which is still reversible senescence [61,62,63]. On the other hand, in late senescence, p53 activity is inhibited and cell senescence is irreversible [62,64]. Primary senescence is paracrine senescence, whereas recently secondary senescence is defined as the process in which primary senescent cells spread senescence via SASP agents and in a juxtracrine way, with direct cell-to-cell contact. Secondary senescence is considered paracrine and juxtacrine senescence together [63,65].

### 3.2. EVs as Biomarkers in Biological and Premature Diseases

In general, aging is a complex process that progressively compromises the biological functions of most organisms. The immune system of the most dysregulated, together with chronic systemic inflammation [66], resulting in increased susceptibility to disease and death. Premature and normal (also known as natural, biological, or physiological) aging share many cellular phenotypes: abnormal nuclear shape, loss of epigenetic markers, immunosenescence (aging immune system), increased reactive oxygen (ROS), and nitrogen species (RNS) that generate lipid, protein, and DNA, increased calcium metabolism, mitochondrial dysfunction, and EV release [67,68,69]. In general, progeronic factors (factors triggering senescence) have been presented in senescent cells during physiological and accelerated aging [70], highlighting an increase in mRNA levels of *p16Ink4a* and *p21Cip1* and the mRNA expression of some cytokines and chemokines is also increased (*Il1β*, *Il6*, *Il10*, *Tnfα Cxcl2*, *Mcp1*, and *Pai1*).

**Figure 3:**
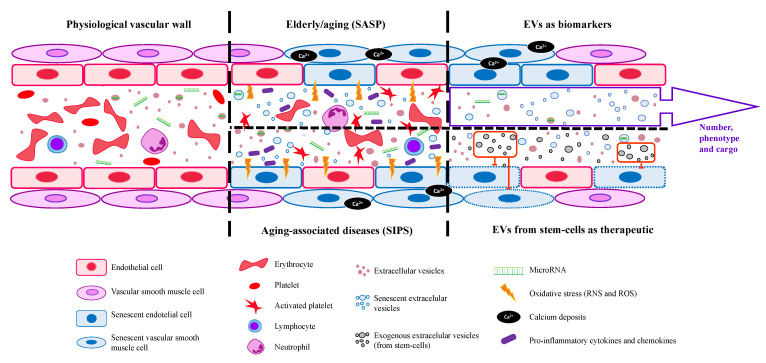
EVs’ general functions in physiological vasculature and aging-associated diseases and their use as a possible biomarker and therapeutic tool. EVs are released by all the body’s cells and their selective cargoes, such as specific miRNAs, may be helpful as biomarkers for age-related disease diagnosis. Moreover, EVs could act as a therapeutic agent for age-associated diseases and the elderly.

Furthermore, protein serum levels of the SASP factors activin A, IL-1β, GDF15, MCP-1, osteopontin (OPN), and beta-2 microglobulin (β2M) were elevated in the serum of aged wild-type mice [71,72]. Cellular senescence is a process characterized by a stable cell cycle arrest [70] that causes inflammation and the capacity to modify the microenvironment (through SASP or SIPS), where cytokines, extracellular matrix proteins, and proteases, as well as other factors that alter the behavior of neighboring cells, have been found driving aging and age-related diseases. Furthermore, Leite et al. [73] identified that an increase in a proinflammatory cytokine, TNF-α, is related to the aging process, confirming the previously published data, in which an association is present between aging and age-related diseases and chronic inflammation [74]. Recently, Boccardi and Mecocci [75] highlighted cellular senescence with advanced age-related diseases and frailty. Figure 3 shows a summary of SASP and SIPS during old age and aging-associated diseases, respectively.

It should be highlighted that EV-mediated processes include cancer progression, immune function, wound healing, and systemic inflammation [16,76]. During inflammation in neurological disorders, the levels of microRNA-155, VCAM1, and ICAM1 increased in small EVs [76]. Thus, senescent EVs cause a loss of homeostasis and can mediate alterations in immunity, inflammation, gene expression, and metabolism of target cells [77]. Moreover, Fafian-Labora et al. have demonstrated for the first time that mesenchymal stem cell-derived EVs have significant age-dependent differences in their immune profiles [78].

In this way, it has been demonstrated that these vesicles play an important role in the aging microenvironment and age-related diseases [48]. An increase in circulating EVs occurs during the progression of aging or aging-associated diseases, whose origin is mainly senescent cells [7]. In general, senescent EVs cause a loss of homeostasis and are mediated by alterations in immunity, inflammation, gene expression, and metabolism of target cells [77]. Due to the transmission of information, EVs present clinical applications: (1) EVs with value as a biomarker focusing on elderly and age-related diseases, and (2) EVs with a potential therapeutic use to extend the lifespan of pathological aging states [7] (Figure 3). Moreover, EVs from senescent cells can transfer the senescence phenotype to the target cells, amplifying the response and the aging environment [7,48]. 

Other review studies have described the role of EVs in some age-associated diseases, including musculoskeletal disorders (osteopenia, osteoporosis, osteoarthritis), neurodegeneration (Alzheimer’s disease, Parkinson´s disease), cardiovascular diseases (vascular calcification, atherosclerosis, cardiac hypertrophy, heart failure, cardiac fibrosis), type 2 diabetes mellitus, lung diseases (chronic obstructive pulmonary disease), and reproductive diseases (ovarian aging and uterine aging) [7,48,79]. We highlight the common point of these studies: the accumulation of senescent cells, which acquire a SASP that contributes to the development and spreading of the disease, partly via releasing EVs. The increased number of EVs and the specific cargo of these EVs have been correlated with the disease progression. For example, the number of EVs obtained from hindlimbs was significantly higher in a mouse model of sarcopenia compared to the age-matched control group, and these EVs were found to be enriched in miR-335-5p, miR-320a, miR-483-5p, and miR-21-5p, both in mice and humans [80]. Evidence suggests that aging modifies EV cargo, particularly the miRNA expression in mice. EVs from aged mice showed an increased expression of miR-146a, miR-21, miR-22, miR-223, miR-145, and let-7a compared to the EVs of young individuals [81].

Consequently, in recent years, the role of microRNA has emerged, including in EVs in the context of aging [19]. Thus, EVs could provide new data on the molecular mechanism of being old and the pathologies associated with premature aging. Furthermore, EVs could be used for diagnostics as a biomarker to prevent aging and aging-related diseases and as a therapeutic strategy to delay old age (Figure 3) [19,82].

Another study suggests that amyloid-β released by microglia in combination with large EVs (Aβ-EVs) moves anterogradely at the axon surface, which contributes to synaptic dysfunction and the spreading of long-term impairment in Alzheimer’s disease [83]. Additionally, EVs released by senescent vascular smooth muscle cells (VSMCs) induced the secretion of IL-10, IL-17, TNF-α, and IFN-γ by T cells and monocytes, thereby suggesting that these EVs influence the cytokine milieu modulating the immune cell activity [84]. Similarly, high levels of inflammatory proteins were found in EVs from diabetic individuals [85]. An in vitro study reported that human mononuclear cells exposed to cigarette smoke become senescent and release EVs enriched in ICAM-1, IL-8, and MCP-1 [86]. Lastly, another study analyzed the follicular fluid-derived EVs obtained from patients with polycystic ovary syndrome and found that these EVs had twenty-five miRNAs that were differently expressed when compared to the control group; in particular, miR-424-5p was found to inhibit granulosa cell proliferation and induce cellular senescence, thereby contributing to the abnormal follicular development during the disease [87]. Taken together, senescent cell-derived EVs are a key player in developing and progressing several age-related diseases.

### 3.3. EVs Cargoes, Concentrations, and Sizes in Aging and Age-Related Pathologies

Like senescent cells, damaged cells tend to release more EVs [88,89,90]. Although EV cargo reflects their parental cell, EV cargo becomes more heterogeneous when derived from damaged or senescent cells instead of healthy cells [63]. Therefore, these cargoes could also be used as biomarkers of aging and age-associated diseases [7,48]. A recent study showed that plasma from young and old mice presents EVs with different cargoes, concentrations, and sizes [7,81]. Thus, aging and its associated diseases can modify the released number of EVs and their cargo, thereby compromising homeostasis [19]. Recently, other studies have shown that particle number and size delivery depend on cell derivation and senescence triggers [7]. Of interest, EV content is heterogeneous and depends on the type of EV and the aging microenvironment. Therefore, due to the ubiquitous presentation or tissue-specific manner, this cargo could also be used as a biomarker of the elderly and aging-associated diseases [7,48]. Accordingly, the heterogenous EV content in aging is a potential biomarker of age-associated diseases in the elderly [7,48]. 

### 3.4. EV Modulation by Environmental Factors

Furthermore, exposure to external/ambient factors, such as air pollution, ultraviolet light, diet, physical exercise, and tobacco, induces changes in the EV profile (exposome). Indeed, an “unhealthy exposome,” defined as a harmful environment, may affect EV composition, thus highlighting how environmental stimuli can predict the incidence of several age-related diseases [91]. Therefore, exposomes could be crucial in bringing changes related to aging and age-related diseases [19]. For instance, it has been suggested that smoking increases EV generation in mononuclear cells [86] and pulmonary cells [92] which leads to an increased production of proinflammatory mediators, such as IL-8, ICAM-1, and MCP-1, thus promoting lung diseases. Similarly, chronic alcohol consumption has been proven to induce the release of EVs enriched with inflammation-related miRNAs, such as miR-30a and miR-192 [19,82,93].

### 3.5. Limitations/Challenges of Using EVs as Biomarkers

EVs are considered a possible biomarker in physiological and pathological processes. In this review, we have described that EVs could be used as a biomarker of biological aging and aging-associated diseases. The reason is that EVs are released by all human cells and are ubiquitous conveyors of intercellular messages and can function as intercellular mediators because of their selective cargoes, such as specific miRNAs, proteins, and lipids [94,95]. They contain a wealth of biomarkers which could be used to monitor the status of some pathologies and clinical conditions [96]. The exact EV contents could be advantageous in using EVs as a biomarker in developing diseases. However, to date, there are some limitations, such as EV isolation and purification, no consensus on the technique to apply, and EV conservation; some difficulties appear in the preservation of samples [94]. The International Society for EVs introduced the state-of-the-art and current challenges in EV-based biomarker discoveries [97].

## 4. Extracellular Vesicles as Therapeutic Agents in Aging and Age-Associated Diseases

In recent decades, EVs have been recognized as VIPs in aging and age-associated diseases. The measurement, quantification, and characterization (selective packaging cargo phenotype) of plasmatic EVs have formed the basis for their potential use in clinical settings and they may be helpful as a biomarker for disease diagnosis and identification of the progression of age-associated diseases and physiological aging [48,98,99]. Additionally, EVs are essential for the evaluation of vesicle-based diagnostics and therapeutic development.

Plasma EVs contribute to the mechanism of aging, transferring their contents (proteins, lipids, and nucleic acids) to cells of all organisms [48]. In fact, EV cargo induces paracrine senescence due to the release of its content, which persists for longer than soluble factors in the target cell [100]. Then, EVs can be postulated as an essential contributor with a geronic role in aging. In this way, it is vital to provide new antiaging therapy. This review shows that EVs from stem cells or young cells containing antioxidative stress machinery [25,101,102] and anti-inflammatory cytokines reduce aging [7].

In an aged context, endothelial EVs present a better antioxidant mechanism to eliminate reactive oxygen species (ROS), antioxidant enzymes, and nicotinamide adenine dinucleotide phosphate (NADPH) [25,101,103]. Accordingly, fibroblast EVs of centenarians contain high levels of antioxidant agents [103]. These data demonstrate that in a senescent environment, cells try to balance the environment affected by DNA damage, inflammation, and all features of senescence, thereby generating an increase in antioxidant agents. However, this is insufficient; the cells remain in a senescent phenotype/status.

### 4.1. Strategies Targeting Senescence

From the therapeutic point of view, senolytic and senomorphic drugs have been developed to regulate cell metabolism by killing senescent cells [104]. Senescence promotes aging and a broad spectrum of age-related diseases, thereby limiting both lifespan and healthspan [55,70,104]. Furthermore, senolytic and senomorphic drugs inhibit the development of some pathologies associated with aging, such as chronic kidney diseases, cardiovascular diseases, osteopenia, and osteoarthritis [55,70,104]. Poblocka et al. demonstrated that using targeted senolytics against specific proteins (surfaceome) in these pathologies could precisely and specifically remove senescent cells and prolong healthspan and lifespan [105]. Another strategy that targets senescent cells is senolysis (removal of senescent cells). Glutaminase 1 (GLS1) expressed by senescent cells keeps them alive, and its inhibition induces senolysis [106]. Therefore, strategies targeting senescence through senotherapy (use of drugs against senescent cells), senolysis (removal of senescent cells), and senoprevention (molecules that inhibit senescence inducers) can delay aging and the development of age-related pathologies, thereby promoting healthy aging [7,69,107,108].

These strategies are emerging therapeutic approaches entering clinical trials [107]. A recent review article summarized the current human clinical trials of senolytic therapies [65]. In another recent review, researchers explained how senolytic therapy effectively decreases human cellular senescence [109]. However, these drugs have unknown long-term effects, which demands caution regarding possible adverse effects [109]. Senescent cells were believed to have resistance to apoptosis. However, Deryabin et al. [110] demonstrated that this is not a general characteristic of senescent cells, and the effect of senolytic drugs targeting cardiac glycosides depends on the status of the apoptosis-prone senescent cells. Despite that, most senolytic agents are used to inhibit antiapoptotic pathways. Suda et al. [57] recently identified non-metastatic melanoma protein B (GPNMB) as a molecular target in senescent cells. GPNMB presents a transmembrane domain considered a seno-antigen that localizes to vascular endothelial cells in humans and mice with atherosclerosis. Mice with apolipoprotein E knockout, a murine model of atherosclerosis (age-related disease), accumulate senescent cells; when these mice were treated with anti-GPNMB, senescence was attenuated, and aging progression was slower than that of unvaccinated mice. Thus, these authors demonstrated that senolytic vaccination can improve the normal and pathological phenotypes associated with aging.

### 4.2. EV as Therapeutic Mediator in Biological and Premature Aging

Similarly, EVs could also act as a therapeutic agent for age-associated diseases and the elderly [48]. Indeed, a recent report demonstrated that EVs can be used as therapeutic agents for skin regeneration due to their anti-inflammatory and immunomodulatory properties [111]. Moreover, EVs promote wound healing by activating migration and proliferation of several skin cell types, such as immune cells, fibroblasts, and keratinocytes, which has been demonstrated in both in vitro and in vivo studies [112,113,114,115,116].

In this sense, a recent EV therapeutical approach mediates processes that shape phenomena such as cancer, immune function, and wound healing [16]. Some studies have shown that EVs from stem cells can delay or inhibit the appearance of senescent cells in aging diseases [7,48]. In the case of premature senescent stem cells, the administration of EVs from healthy cells can improve cell function and stemness [55]. Furthermore, EVs from mesenchymal stem cells can inhibit the translocation of the nuclear factor kappa-B (NF-κB) transcription factor p65 to the nuclei, an inflammatory factor activated in SASP, which prevents aging [117]. A recent study reported that EVs secreted mainly from stem cells (adipose tissue and bone marrow-derived mesenchymal stromal cells) can increase angiogenesis and osteogenesis because they contain a higher amount of prodifferentiation and chemotactic proteins [118]. This has also been demonstrated in a proteomic study of EVs derived from mesenchymal stromal cells [119]. The authors demonstrated the possibility of modifying the EV phenotype through changes in parental cells, thereby confirming that EVs from stem cells are a potential therapy for degenerative and immunological diseases.

Moreover, another study reported that EVs obtained from mesenchymal stromal cells can modulate the immune response in the progression of neurodegenerative conditions that lead to premature aging and death [120]. More studies have demonstrated that EVs are critical in aging and aged-linked diseases. Some examples are: Ly6G+ plasma EVs were associated with improved fracture healing in aged mice of heterochronic parabiosis pairs [121] and the case of EV cargo of human embryonic stem cells (hESCs) and human-induced pluripotent stem cells (hiPSCs) with antiaging potential [122].

A recent study showed that human mesenchymal stroma-/stem-like cell exosome treatment reduced renal tubular cell senescence, thereby delaying the development of renal aging [123]. These studies highlight that mesenchymal stromal cell-derived EVs could act as an innovative therapeutic tool for regenerative medicine in aging-related diseases [17,124]. Therefore, EVs from stem cells can be potentially used as a therapeutic vehicle in senotherapy owing to their regenerative potential.

The first evidence of therapeutic use (clinical phase 1 trial) of EVs from mesenchymal stromal cells was in a patient who suffered from Menière’s disease, a neurodegenerative disorder. EVs were intracochlearly administered, and the results showed that EVs presented a clinical benefit by acting as a local adjuvant treatment [125].

EV-derived miRNAs are crucial regulators of age-related diseases. Some miRNAs, such as miRNA-21, miRNA-29, and miRNA-34, are regulators of regeneration and functionalities of different tissues, and they are implied in physiological and physiopathological signaling, which affect life expectancy [99]. The human miRNA profiles of young and elderly individuals are different [99]. Therefore, miRNAs derived from EVs are critical for the regulation of the aging process. Moreover, a senolytic treatment reversed the EV content pattern (miRNA) in aging mice and provided a more youthful phenotype [81]. Another study reported that senolytic drugs in aged mice generated EVs with different miRNA cargo, thereby reducing pain and degeneration in osteoarthritis disease [126]. These studies confirm that EV content can be potentially used in the diagnosis of aging diseases, and they can be used as a therapeutic target to measure the efficacy of senolytic treatments. 

Aging (physiological or premature) declines the body’s function, which is highlighted by the gradual degradation of muscles, which become smaller, weaker, and susceptible to delayed reparation after injury. In this context, a recent study demonstrated that plasma EVs from young mice contained Klotho (a longevity protein), and administering these EVs to aged animals with injured muscles accelerated muscle regeneration capacity [127]. The authors proposed using EVs as a regeneration booster for older individuals.

## 5. Conclusions

In recent decades, the concept of EVs has changed. When they were initially discovered, EVs were cellular dust; therefore, they did not have any functions; with time, this concept has changed and will probably continue being updated. Nowadays, EVs are considered critical mediators in physiological and pathophysiological processes. Therefore, this study summarizes the current knowledge on EVs from their discovery as cellular dust to their recognition as “very important particles” (VIPs) that mediate cell–cell communication and the current and newest isolation methods. Moreover, we describe the role of EVs in aging and age-related diseases and their potential use in the clinic as biomarkers for early diagnosis and as therapeutic agents for disease treatment.

EVs display several features that provide them with invaluable abilities for their application in regenerative medicine. First, the EV content mimics their parental cells. Thus, isolation of EVs from body fluids with a specific cargo is very useful as a biomarker in disease development for early diagnosis. Second, EVs have a specific set of surface proteins that indicates their target cells. This characteristic also has a valuable potential for their use as drug-delivery carriers to target cells. To date, EVs are VIPs because they act as mediators in physiological and pathological processes. Particularly, as shown in this review, EVs mediate biological aging and premature aging by their content and number released by senescent cells. 

Research is still needed in the EV therapeutic field, in particular, focusing on the development of autologous EVs that would enable personalized treatment for each specific disease. Regarding the research on EV-mediated mechanisms of aging, efforts should be performed to establish a time- and cargo-dependent correlation between EVs and the incidence of age-related diseases so that EVs become a very early biomarker. It is also necessary to elucidate the exact molecular mechanisms involved in the change in EV content during aging; this understanding would help us to develop new cell-free treatments to reverse age-related diseases in the future. However, it should be mentioned that before starting antiaging therapies with EVs, safety, sensitivity, and specificity must be precisely verified. Additionally, administration, dosages, treatment intervals, and duration must be strictly certified.

**Figure 4:**
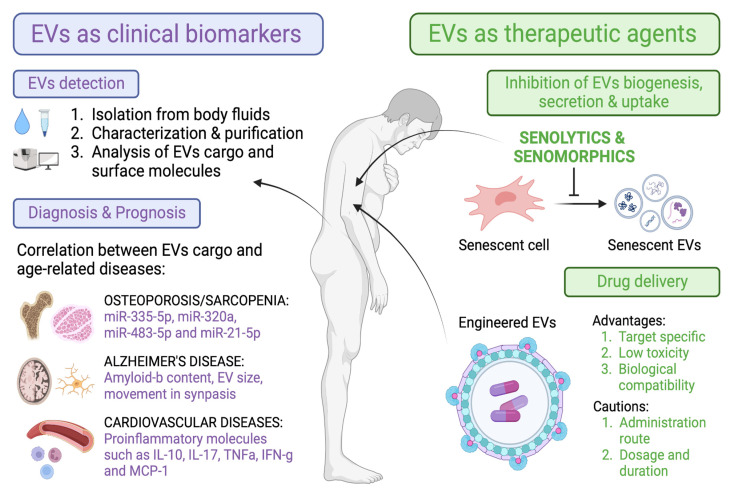
Overview of the main characteristics of EVs that make them suitable tools as biomarkers and therapeutic agents in aging and age-related diseases.

As a take-home message, our main aim in this review was to summarize the different roles of EVs described in the literature as novel biomarkers of age-related diseases, as well as the potential use of different senotherapies (senolytics or senomorphics) to modulate EVs (shedding, cargo content, and uptake) and thereby the spreading and progression of the disease (Figure 4).

## 6. Contribution to the Field Statement

Aging- and premature aging-associated diseases are considered chronic inflammatory diseases that degrade the quality of life and can lead to death. In this context, EVs are VIPs because they are crucial for the molecular mechanisms associated with the development of these types of illnesses, which deteriorate body homeostasis. Therefore, the characterization of EVs is essential because they act as biomarkers of aging diseases and can be potentially used as therapeutic agents to delay aging and prevent age-associated diseases.

## Figures and Tables

**Table 1:** Advantages and disadvantages of EV quantification and characterization methods.

Method	Quantification	Characterization	Advantages	Disadvantages	References
Flow cytometry (FC) or flow cytometry experiment on extracellular vesicles (MIFlowCyt-EV)	Yes	Yes (specific surface cell origin marker)	Characterization of particles with count rate 2–4 orders of magnitude higher than other techniques More accurate and precise concentration and particle diameter Used to phenotype fluorescently labeled EVs Commonly available in clinical laboratories Reliable determination of particle diameter as the optical properties of particles can vary greatly	Detection limit of conventional FC equipment (diameter < 300 nm) and new flow cytometry models (~200 nm) FC detects single cells rather than single EVs. Standardization of EV FC affects: (1) EV optical properties, (2) complexity of EV-containing samples, (3) differences between FCs EV size distribution in plasma is non-normal, with a peak <200 nm and a long tail pointing toward larger diameters Differences between FC affect the standardization of EV measurements	[2,39,44]
Nanoparticle tracking analysis (NTA)	Yes	Yes	Size distribution of particles measured in EV isolation Measurement of the distribution of absolute size of vesicles from 50 nm to 1 μm. New NTA can measure up to 2 μm “NTA fluorescence” (NTA-FL) detects EV protein markers from cell origin	Small particles (<60 nm) not detected (sensitivity limit of NS3000 is 46–70 nm) Overestimation of EV amount owing to the presence of lipoproteins and protein aggregates highlights limitations, particularly in human serum NTA cannot distinguish EVs from particles of the same size in suspension (debris). New NTA is associated with fluorescence to distinguish EVs from debris and proteins	[2,38,39,45,46,47]
Western blot (WB)	No	Yes (example: CD63 protein = exosome markers)	No universal EV marker, depending on the kind of EVs and their origin cell	Proteins (example: CD63) detected by WB are low but tend to correlate with the amount of protein in the sample	[39]
Transmission electron microscopy (TEM)	No	Yes (size and morphology)	Visualization of cup-shaped EVs with compatible morphology and size	Low quantification of EVs per field No characterization of EVs	[38,39]
Dynamic light scattering (DLS)	No	Yes (range distribution)	Size distribution of vesicles from 1 nm to 6 μm	Absolute concentration of vesicles cannot be determined because the average amplitude of the signal cannot be determined It depends on the diameter, concentration, and refractive index of EVs	[2,38]
Tunable resistive pulse sensing (tRPS)	Yes	No	EVs move through tunable nanopores, which can register EVs of 80–1000 nm. The size currently detected by tRPS is superior (up to 5700 nm depending on the nanopore)	It cannot distinguish between EVs and similarly sized particles	[2,38,45]
High-resolution flow cytometry (hFC)	Yes	Yes	Sensitive and robust method for analysis of fluorescently labeled EVs and small EV subpopulations (human plasma samples and cell culture-derived samples) Small EV subpopulations stained with different fluorescent antibodies can be analyzed in a multiparametric manner	hFC does not allow for absolute size measurement of EVs	[38,45,48]
ExoView	Yes	Yes	Single EV analyses EV size and phenotypic analyses Direct analysis of EVs in biofluids Its ability to visualize smaller particles and detect targets with low expression levels Discrimination between sEV and contaminants, such as bovine-specific sEV Detection limit: 50 nm	Background signal in tetraspanin profile	[49,50,51]
